# Staphylococcus aureus Redirects Central Metabolism to Increase Iron Availability

**DOI:** 10.1371/journal.ppat.0020087

**Published:** 2006-08-25

**Authors:** David B Friedman, Devin L Stauff, Gleb Pishchany, Corbin W Whitwell, Victor J Torres, Eric P Skaar

**Affiliations:** 1 Mass Spectrometry Research Center, Department of Biochemistry, Vanderbilt University Medical Center, Nashville, Tennessee, United States of America; 2 Department of Microbiology and Immunology, Vanderbilt University Medical Center, Nashville, Tennessee, United States of America; Harvard Medical School, United States of America

## Abstract

Staphylococcus aureus pathogenesis is significantly influenced by the iron status of the host. However, the regulatory impact of host iron sources on S. aureus gene expression remains unknown. In this study, we combine multivariable difference gel electrophoresis and mass spectrometry with multivariate statistical analyses to systematically cluster cellular protein response across distinct iron-exposure conditions. Quadruplicate samples were simultaneously analyzed for alterations in protein abundance and/or post-translational modification state in response to environmental (iron chelation, hemin treatment) or genetic *(Δfur)* alterations in bacterial iron exposure. We identified 120 proteins representing several coordinated biochemical pathways that are affected by changes in iron-exposure status. Highlighted in these experiments is the identification of the heme-regulated transport system (HrtAB), a novel transport system which plays a critical role in staphylococcal heme metabolism. Further, we show that regulated overproduction of acidic end-products brought on by iron starvation decreases local pH resulting in the release of iron from the host iron-sequestering protein transferrin. These findings reveal novel strategies used by S. aureus to acquire scarce nutrients in the hostile host environment and begin to define the iron and heme-dependent regulons of S. aureus.

## Synopsis

Virtually all bacterial pathogens require iron to successfully infect their human hosts. This presents a problem to invading bacteria because the majority of iron in humans is tightly bound by iron-binding proteins. To counteract this host defense, bacterial pathogens have developed elaborate mechanisms to acquire nutrient iron during infection. To gain insight into how the amount of available iron impacts the human pathogen *Staphylococcus aureus,* the authors identified proteins that increase or decrease abundance upon alterations in iron status. The authors found that under conditions of iron starvation, the Fur regulatory protein of S. aureus coordinates a redirection of the central metabolic pathways causing the bacteria to produce large amounts of acidic end-products. The accumulation of these acidic end-products facilitates the release of iron from host iron-binding proteins, in effect increasing the availability of this precious nutrient source. These findings provide a mechanistic explanation for how S. aureus alters its local microenvironment during infection to increase the availability of nutrient iron. Based on the well-established role for bacterial iron acquisition during pathogenesis, systems involved in iron acquisition represent excellent potential therapeutic targets against bacterial infection.

## Introduction

Staphylococcus aureus requires iron to successfully colonize the host [1]. To ensure efficient uptake and metabolism of host iron sources, bacterial pathogens regulate a variety of genes in response to the levels of available iron. The canonical bacterial repressor responsible for this iron-dependent regulation is the ferric uptake regulator (Fur) [2]. S. aureus has a functional Fur which has been implicated in the iron-dependent repression of a subset of genes [3–5]. An *S. aureus Δfur* mutant has a significant defect in virulence in a mouse model of infection, underscoring the importance of iron metabolism to staphylococcal pathogenicity [6]. The consensus sequence to which the S. aureus Fur binds has been predicted using in silico techniques [7]; however, a global analysis of Fur and iron-affected proteins in this important human pathogen has not been reported. The demonstrated role for iron and Fur in staphylococcal pathogenesis emphasizes the importance of identifying the iron-dependent Fur regulon of S. aureus.

Heme is the preferred iron source of *S. aureus,* and heme acquisition contributes to staphylococcal infection [8]. We have proposed a model for heme-Fe acquisition that involves the hemolysin-dependent lysis of host erythrocytes followed by hemoglobin recognition, heme removal, and transport into the bacterial cytoplasm [9]. Once inside the bacterium, heme can either be degraded by staphylococcal heme monoxygenases [10,11] or segregated to the bacterial membrane, where it is likely incorporated intact into bacterial heme-binding proteins [8]. It is possible that the different potential fates for intracellular heme are dependent on the level of iron and/or heme exposure experienced by the bacterium in distinct host environments. If correct, this model suggests that bacterial pathogens monitor the level of intracellular heme and alter protein expression in response to changes in heme status.

Based on the demonstrated role for iron, Fur, and heme in staphylococcal pathogenesis, we sought to evaluate changes in global protein status in response to alterations in bacterial iron status using two-dimensional (2D) difference gel electrophoresis (DIGE). DIGE enables quantitative differential-display analysis with statistical confidence and is based on 2D gel separations whereby thousands of protein features can be resolved based on isoelectric point and by apparent molecular mass. It uses spectrally resolvable fluorescent dyes (Cy2/3/5) to prelabel samples that are then multiplexed onto the same gel, allowing for direct quantification of each resolved protein feature between the three dye channels without analytical (gel-to-gel) variation. Multiple samples from a complex experiment can be analyzed across several DIGE gels, whereby an internal standard comprised of every sample present in the experiment is included in each multiplexed gel [12–15]. Within each gel, quantitative measurements are made for each resolved protein feature relative to the cognate signal from the internal standard, which is then used to normalize the intragel ratios between gels in a coordinated experiment. Thus, DIGE enables multiple conditions with repetition to be quantitatively analyzed with statistical confidence. Proteins of interest are then identified using mass spectrometry (MS) and database interrogation. Multivariate algorithms, such as principle component analysis (PCA) and unsupervised hierarchical clustering, can now be applied to DIGE datasets to enable analysis of global expression patterns. Combining multivariable DIGE/MS with multivariate statistical analyses clusters cellular protein responses across distinct environmental conditions based on total expression profiles. These technologies can be combined to identify expression changes in coordinated biochemical pathways.

In this manuscript, we report the application of multivariable DIGE/MS to S. aureus cultures exposed to various biochemical and genetic manipulations in cellular iron status. We found that 21 distinct proteins undergo expression changes in response to exogenous hemin, representing the first reported global analysis of bacterial proteins that are affected by this host molecule. Further, through biochemical classification of proteins undergoing expression changes upon alterations in iron status, we observed an overrepresentation of proteins involved in the central metabolic pathways of S. aureus. Based on these observations, a series of experiments was performed revealing that the Fur protein of iron-starved S. aureus redirects central metabolic pathways to increase production of lactate as a fermentative end-product. This increase in lactate production contributes to a decrease in local pH, facilitating the release of iron from host transferrin. Results obtained from this study identify a novel strategy used by S. aureus to increase host iron availability and begin to define the iron- and heme-dependent regulons of S. aureus.

## Results

### Changes in Iron Status Alter Staphylococcal Protein Expression Patterns

**Figure 1:**
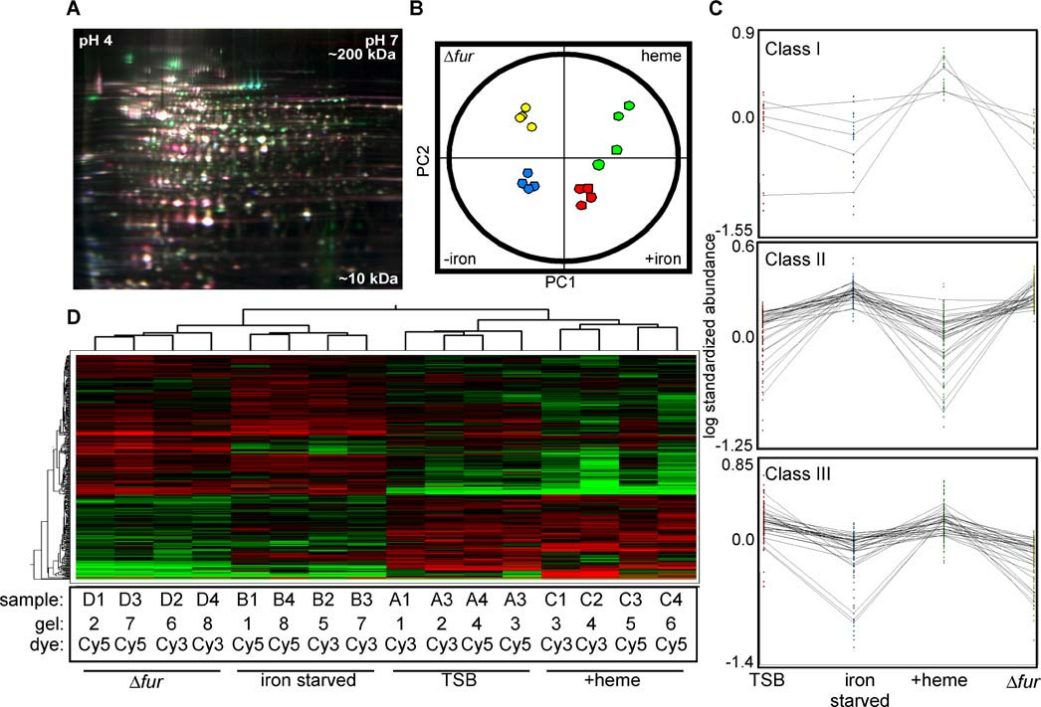
Proteome Analysis Using 2D DIGE (A) False–colored representative gel from the eight-gel set containing three differentially labeled samples as described in Materials and Methods. Cy2-labeled internal standard (blue), Cy3-labeled control No. 1 (green), and Cy5-labeled iron-starved No. 1 (red) are overlaid for illustrative purposes. (B) Unsupervised PCA properly groups the 16 individual DIGE expression maps differentiated by two principle components (PC1 and PC2) and demonstrates high reproducibility between the replicate samples within each group. (C) Composites of DIGE expression patterns representing the five proteins that increase abundance in the presence of hemin (Class I, Table 1), the 29 Class II protein features negatively affected by Fur and iron (Table 2), and the 30 Class III protein features positively affected by Fur and iron (Table 3). (D) Unsupervised hierarchical clustering of the 16 individual DIGE expression maps (groups, shown along top) and of individual proteins (shown on the left), with relative expression values for each protein displayed as a heat map using a relative scale ranging from −0.5 (green) to +0.5 (red). The gel number (1 through 8), samples (A, TSB; B, iron-starved; C, hemin; and D, *Δfur*), and Cy3/5 dye labeling for each sample are listed below.

To identify proteins that are affected by alterations in host iron sources, we performed differential expression analyses on S. aureus cultures grown under various conditions of iron exposure. Cytoplasmic proteins were prepared from wild-type and *Δfur* mutant cells grown under either iron-replete conditions, after iron starvation elucidated by treatment with 2,2′-dipyridyl (DIP), or after exposure to hemin. Protein extracts from each of the four conditions were independently isolated in quadruplicate to control for nonbiological variation, and the resulting 16 extracts were simultaneously coresolved across eight DIGE gels that were coordinated by a Cy2-labeled 16-mix pooled-sample internal standard as described in Materials and Methods (Figure 1). PCA was used to group the 16 individual Cy3- or Cy5-labeled proteome maps based on the overall expression pattern from the more than 1,000 resolved protein forms under survey. PCA allowed for independent confirmation of distinct expression patterns from the four groups and demonstrated high reproducibility between the replicate samples. The four groups (control, iron-starved, *Δfur,* and hemin) all clustered into separate quadrants with only one proteome map from the hemin group clustering equidistant from the hemin group and the control group (Figure 1B). These assignments were reiterated in an unsupervised hierarchical clustering of the independent proteome maps presented as a heat map, where expression patterns of the individual proteins can also be compared (Figure 1D).

**Table 1:**
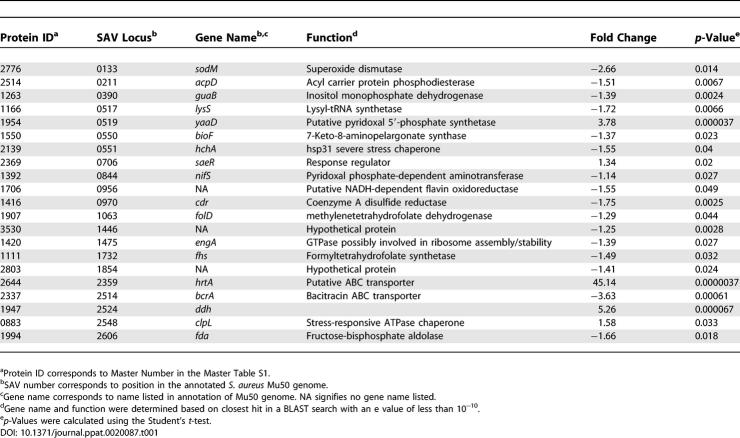
Proteins Affected by Hemin Independent of Iron and Fur

**Table 2:**
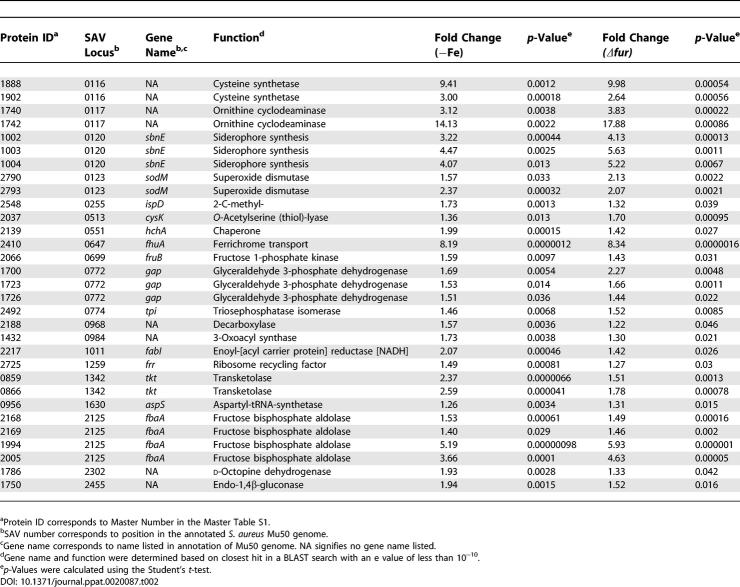
Proteins Negatively Affected by Fur and Iron

**Table 3:**
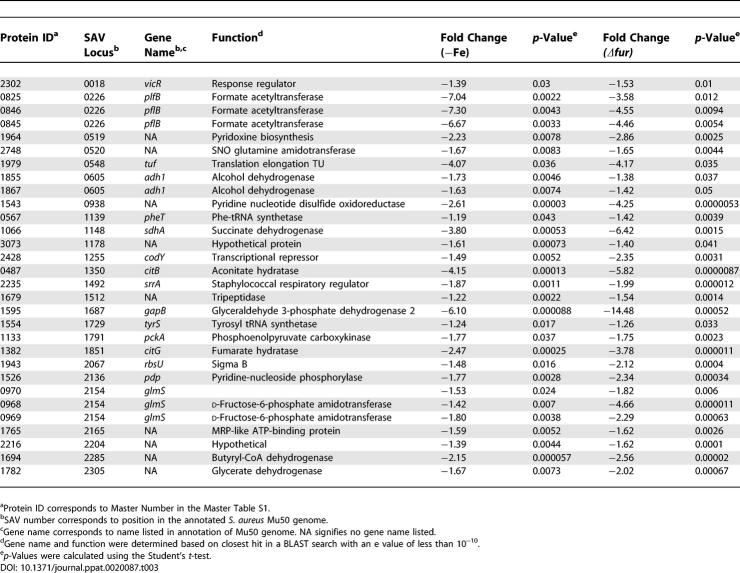
Proteins Positively Affected by Fur and Iron

DIGE analysis combined with subsequent PCA and hierarchical clustering allowed for the grouping of protein expression changes between these four conditions into the following five classes: (I) proteins that are affected by hemin independent of Fur or iron (Table 1, proteins that are more abundant as shown in Figure 1C), (II) proteins that are negatively affected by iron and Fur (Table 2 and Figure 1C), (III) proteins that are positively affected by iron and Fur (Table 3 and Figure 1C), (IV) proteins that are affected by iron independent of Fur (Table 4), and (V) proteins that are affected by Fur independent of iron (Table 5). We identified 29 resolved protein features (representing 20 distinct proteins including isoforms) under iron-dependent negative control by Fur, 30 distinct features (25 proteins including isoforms) under iron-dependent positive Fur-mediated control, and 21 distinct proteins that are exclusively affected by hemin.

**Table 4:**
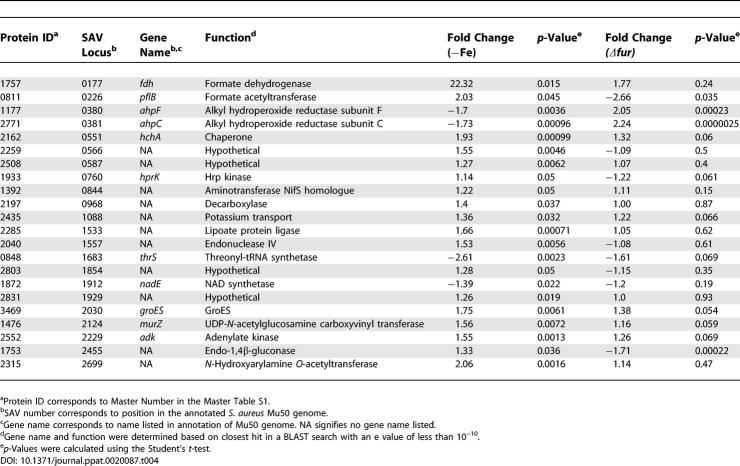
Proteins Affected by Iron Independently of Fur

**Table 5:**
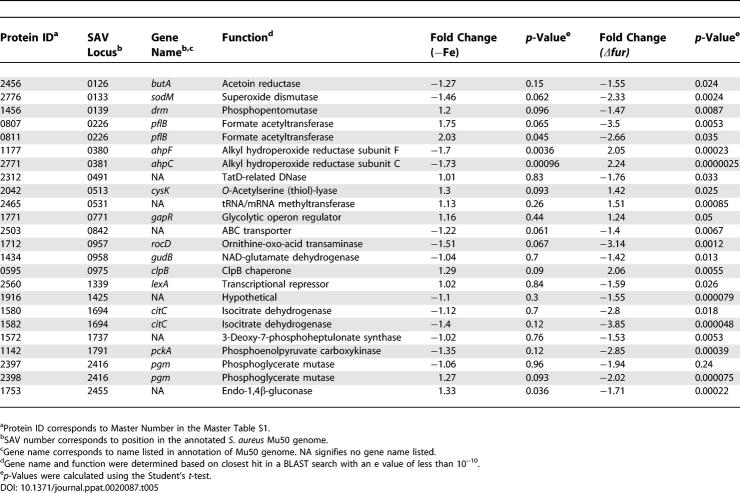
Proteins Affected by Fur Independently of Iron

Using the PCA and hierarchical clustering approach, we were able to further group the proteome expression maps into two primary clusters, one containing all eight samples prepared from bacteria grown in TSB or TSB containing hemin and a second containing all eight samples prepared from *Δfur* bacteria or bacteria starved for iron. This comprised the first principle component, which accounted for 62.3% of the variance in the system. These multivariate analyses of protein expression changes allow for a global representation of the similar patterns in protein expression that occur upon inactivation of *fur* versus those that occur upon iron starvation. Furthermore, this analysis reveals that exposure of S. aureus to hemin results in a vastly different, and less severe, change in cellular protein expression as compared to altering the iron status of the bacterium (Figure 1D).

### S. aureus Proteins Regulated by Hemin Independently of Iron or Fur (Class I)

The reactive nature of heme presents a unique problem to bacterial pathogens, as they must maintain an intracellular heme homeostasis that prevents toxicity while internalizing enough heme for nutrient iron needs [8]. This raises the possibility that bacterial pathogens undergo a coordinated change in protein expression in response to exogenous heme. To identify proteins that respond to heme, we grew *S. aureus* in the presence of 10 μM hemin and identified proteins that change expression upon hemin exposure but not upon inactivation of *fur* or iron starvation. Twenty-one proteins were identified that responded exclusively to excess hemin with statistical confidence (0.04 > *p* > 0.0000037), 16 of which were down-regulated between 1.25-fold and 3.6-fold (Table 1). These proteins represent a variety of predicted biochemical functions without clear overrepresentation of any one physiological pathway. Only five proteins increased expression exclusively in response to hemin, comprising a hemin-activated regulon (Figure 1C). These proteins are involved in lactate metabolism (Ddh, 5.26-fold, *p* = 0.000067), gene regulation (SaeR, 1.34-fold, *p* = 0.02), and stress response (YaaD, 3.78-fold, *p* = 0.000037, ClpL, 1.58-fold, *p* = 0.033). The fifth protein, a putative conserved ABC transporter (SAV2359), exhibited a 45-fold increase (*p* = 0.0000037) upon exposure to hemin, and is predicted to encode for a conserved transporter with no demonstrated function. These experiments describe the first global analysis of the heme-regulon of a bacterial pathogen and identify a putative transport system that is highly up-regulated exclusively upon exposure to hemin.

### The Hrt System Is Required for S. aureus Growth in Hemin

The protein demonstrating the most significant increase upon hemin exposure (45-fold increase) is an ATP-binding component (SAV2359) of a previously uncharacterized ABC-type transport system. The gene encoding for SAV2359 is located immediately adjacent to a predicted permease component (SAV2360) of the same transport system. Based on our proteomic observations, we have named these proteins the heme-regulated transporter ATPase (HrtA) and permease (HrtB). Importantly, genomic analyses demonstrate that this transport system is conserved across many pathogenic bacteria, including *Bacillus anthracis, Listeria monocytogenes,* and *Enterococcus faecalis* (unpublished data).

**Figure 2:**
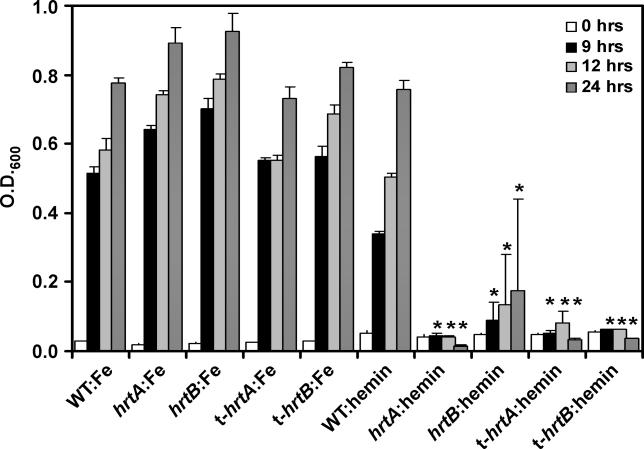
The Hrt System Is Required for Staphyloccocal Growth in Hemin S. aureus Newman (WT), SAV2359 mutant *(hrtA),* SAV2360 mutant *(hrtB),* transduced SAV2359 mutant *(*t-*hrtA),* and transduced SAV2360 mutant *(*t-*hrtB)* strains were grown in iron-free medium supplemented with iron (Fe) or with hemin (hemin). Bacterial growth was determined by measuring the optical density (O.D._600_) of cultures. Results represent the mean ± SD from triplicate determinations. Asterisks denote statistically significant differences from wild-type as determined by a Student's *t*-test (*p* < 0.05).

To explore the contribution of the Hrt system to heme transport, we investigated whether strains inactivated for *hrtA* or *hrtB* can grow when hemin is the sole available iron source. There was no detectable difference in the ability of wild-type, *hrtA,* and *hrtB* mutant strains to grow in medium where the sole iron source was FeSO_4_ (Figure 2). In contrast, compared to wild-type, the *hrtA* and *hrtB* mutant strains are severely impaired in their ability to grow when hemin is the sole iron source (Figure 2). To further confirm that the hemin-dependent growth defect exhibited by strains inactivated for *hrtA* and *hrtB* is dependent on the insertional mutations, and as an attempt to rule out the possibility that the observed growth defects were due to secondary mutations, we transduced the *hrtA* and *hrtB* mutations into a clean wild-type background as previously described [10]. Successful transductants exhibited identical phenotypes as the original *hrtA* or the *hrtB* mutants, suggesting that inactivation of the Hrt system is responsible for the observed inability to grow in the presence of high hemin concentrations (Figure 2). Taken together, these observations identify the HrtAB as a novel staphylococcal heme transport system that is critically important to proper heme metabolism.

### S. aureus Proteins Negatively Regulated by Iron and Fur (Class II)

Proteins that increase abundance upon iron starvation or inactivation of *fur* (via release from repression) represent proteins negatively regulated by Fur in an iron-dependent manner, and hence comprise the canonical Fur regulon of the bacterium. We identified 29 distinct protein features comprising 20 unique cytoplasmic proteins that are increased from 1.3-fold to over 9-fold in the absence of iron or Fur (0.04 > *p* > 0.0000012, Table 2). These results demonstrate a strong correlation between expression changes upon iron chelation versus the absence of Fur. As expected, iron acquisition systems previously shown to be iron-regulated via Fur are up-regulated under these conditions including proteins involved in siderophore synthesis (SbnE isoforms exhibiting over 3-fold increases in minus iron or *Δfur,* 0.013 > *p* > 0.00013) [16] and transport (FhuA, over 8-fold increases, *p* < 0.0000016) [17].

**Figure 3:**
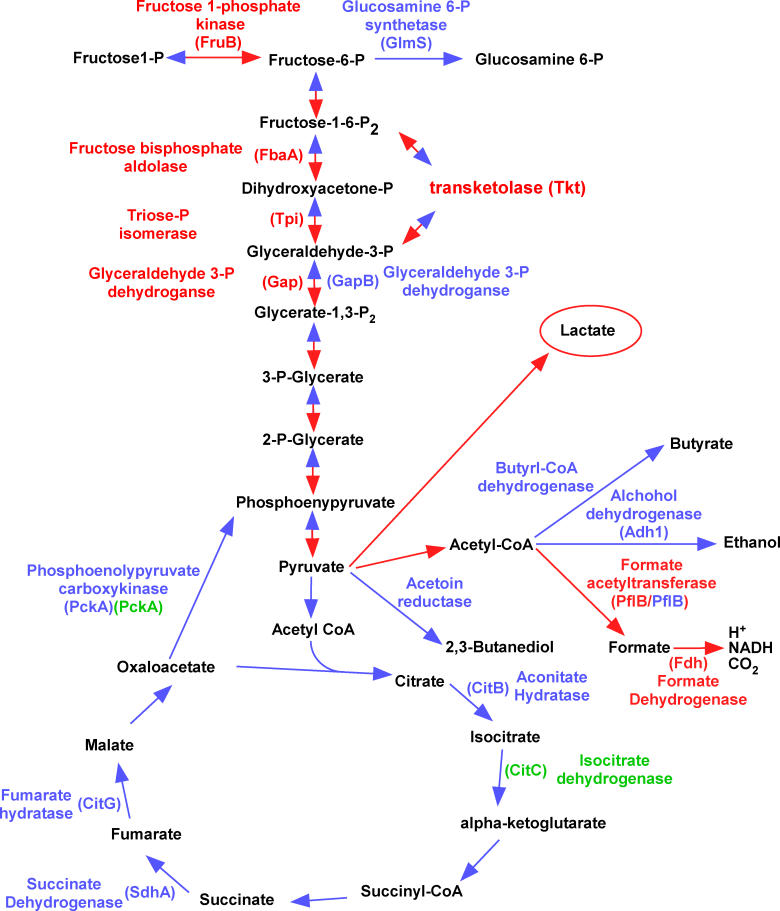
Lactate Is a Primary End-Product of Carbohydrate Metabolism by Iron-Starved S. aureus A subset of the predicted central metabolic reactions of S. aureus is shown. Proteins shown in red are up-regulated in the absence of iron or upon inactivation of *fur*. Proteins shown in blue are down-regulated in the absence of iron or upon inactivation of *fur*. Proteins shown in green are down-regulated in the absence of Fur independent of iron status. The red arrows predict the direction of reactions upon iron starvation, while the blue arrows predict reactions that are inhibited upon iron starvation.

Five of 21 proteins that were classified as having Class II expression patterns with mostly moderate increases (approximately 1.5-fold) are enzymes of the glycolytic pathway including fructose 1-P kinase (FruB), fructose bisphosphate aldolase (FbaA), triosephosphate isomerase (Tpi), glyceraldehyde 3-phosphate dehydrogenase (Gap), and transketolase (Tkt) (Table 2). This observation is consistent with a systemic up-regulation of glycolysis upon iron starvation, which would lead to a commensurate increase in pyruvate for subsequent use in the tricarboxylic acid (TCA) cycle or as a substrate for fermentative metabolism (Figure 3).

### S. aureus Proteins Positively Affected by Iron and Fur (Class III)

Fur is traditionally considered a repressor of iron-regulated gene transcription. However, recent work has highlighted a role for Fur in the direct and/or indirect activation of a small subset of genes in Helicobacter pylori [18], Vibrio cholerae [19], Neisseria meningitidis [20], Escherichia coli [21,22], and Bacillus subtilus [23]. We identified 30 distinct protein features representing 25 unique proteins that were positively affected by Fur in an iron-dependent manner as measured by decreased detection in the absence of Fur or upon iron starvation (Table 3).

Numerous regulatory factors were positively affected by Fur in an iron-dependent manner including RsbU. RbsU, down 2.12-fold (*p* = 0.0014) and 1.48-fold (*p* = 0.016) in *Δfur* and iron-deplete conditions, respectively, controls the expression of a variety of virulence genes and regulatory systems. Based on this pleotropy, small changes in RbsU expression may have profound affects on cellular metabolism. In particular, RbsU activates acetate catabolism; therefore, the Fur-dependent activation of RbsU is consistent with a down-regulation of the TCA cycle upon iron starvation or *fur* inactivation [24].

The value of iron to the bacterium is underscored by the Fur-mediated iron dependent increase of four separate proteins that are predicted to contain iron-sulfur clusters. Three of these proteins are the TCA cycle enzymes succinate dehydrogenase (SdhA, decreased 6.42-fold in *Δfur, p* = 0.0015 and 3.8-fold in iron-depleted, *p* = 0.00053), aconitate hydratase (CitB, decreased over 4-fold, *p* < 0.0002 in both conditions), and fumarate hydratase (CitG, decreased over 2-fold in both conditions, *p* < 0.00025). A fourth TCA cycle enzyme, phosphoenolypyruvate carboxykinase (PckA), which coverts oxaloacetate to phosphoenypyruvate during gluconeogenesis, was decreased approximately 1.7-fold upon inactivation of *fur* (*p* = 0.0023) or iron depletion (*p* = 0.037). Two additional proteins associated with central metabolism and demonstrating group III expression patterns are d-fructose-6-phosphate amidotransferase (GlmS) exhibiting 2.29-fold (*p* = 0.00063) and 1.8-fold (*p* = 0.0038) decreases in *Δfur* and iron-depleted conditions, respectively, and glyceraldehyde 3-P dehydrogenase (GapB), exhibiting 14.5-fold (*p* = 0.00052) and 6.1-fold (*p* = 0.000088) decreases in *Δfur* and iron-deplete conditions, respectively. GlmS converts fructose 6-P to glucosamine-6-P, and hence depletes substrate for phosphofructokinase in effect antagonizing glycolysis. GapB is a second glyceraldehyde 3-P dehydrogenase of S. aureus and based on its function in *Bacillus subtilus,* is predicted to possess an enzymatic GAPDH activity involved in gluconeogenesis [25]. These results support the Fur-mediated up-regulation of glycolysis upon iron starvation and suggest a commensurate systemic and regulated inhibition of the TCA cycle. Together, these findings support a model whereby in iron-starved *S. aureus,* excess pyruvate produced as a result of an up-regulation of the glycolytic pathway is shuttled into fermentative pathways as opposed to the TCA cycle.

In keeping with the above model, Class III proteins associated with fermentative metabolism were represented by alcohol dehydrogenase (Adh1), butyryl-CoA dehydrogenase (between 1.5- to 7-fold decreases for the two conditions, 0.05 > *p* > 0.00002), and three distinct isoforms of formate acetyltransferase (PflB) (between 3.6 to 7.3-fold decreases for the two conditions across isoforms). These three enzymes are involved in the conversion of pyruvate to distinct end-products of fermentative metabolism: formate, ethanol, or butyrate, respectively. These decreases upon iron chelation suggest that iron-starved S. aureus metabolize pyruvate through fermentation to metabolic end-products other than formate, ethanol, or butyrate. These results suggest that excess pyruvate produced as a result of increased glycolysis is converted to other predicted products of staphylococcal fermentative metabolism, such as 2,3-butanediol and/or lactate (Figure 3).

### S. aureus Proteins Regulated by Iron Independently of Fur (Class IV)

In addition to Fur, S. aureus possesses the metal-dependent regulators Zur [26], PerR [27], and MntR [28]. The presence of multiple metal-specific regulatory factors raises the possibility that additional as-yet-unidentified factors other than Fur respond to changes in cellular iron content. We identified 22 unique proteins that were positively or negatively affected by iron starvation, but whose expression was not affected by inactivation of *fur* (Table 4). Four of these proteins are associated with cellular respiration including formate dehydrogenase (Fdh) which was up-regulated 22.32-fold in the absence of iron (*p* = 0.015), Hpr Kinase (HprK), NAD synthase (NadE), and a single isoform of formate acetyltransferase (PflB). Thus, we have identified a significant pool of proteins that are affected by iron independently of Fur, raising the possibility that an additional transcriptional regulator exists in S. aureus to monitor intracellular iron status*.* It should be pointed out that DIP binds divalent cations other than iron which might be responsible for some of the Class IV expression changes that were observed.

### S. aureus Proteins Regulated by Fur Independently of Iron (Class V)

We also identified 24 unique proteins that changed expression upon inactivation of *fur* without any significant changes in iron availability status (Class V, Table 5). One Class V protein, GapR, is an activator of Gap expression and is increased in the absence of Fur (1.24-fold, *p* = 0.05). This moderate increase in expression may contribute to the increase in Gap expression observed upon inactivation of *fur* (as much as 2.27-fold, *p* = 0.0048; Table 2). Inactivation of *fur* leads to down-regulation of PckA (2.85-fold, *p* = 0.00039) and isocitrate dehydrogenase (CitC; 3.85-fold, *p* = 0.0018), consistent with our previous observation of Fur-mediated activation of the TCA cycle. Furthermore, acetoin reductase (ButA) is decreased by inactivation of *fur* (1.55-fold, *p* = 0.024) implying a commensurate decrease in the production of 2,3-butanediol upon iron starvation. Taken together with results described above, this suggests that a major metabolic end-product of carbohydrate metabolism produced by iron-starved S. aureus is lactate (Figure 3).

### Iron-Starved S. aureus Produce Excess Lactate Leading to a Decrease in Local pH

Iron-starved S. aureus are known to restrict oxidative capacity by oxidizing glucose with the accumulation of much lactate and minor amounts of pyruvate, acetate, and acetoin [29]. The coordinated expression changes of the staphylococcal central metabolic pathways identified using DIGE/MS are summarized in Figure 3 and provide a mechanistic explanation for this observation. Our results suggest that iron-starved S. aureus undergo a Fur-mediated redirection of central metabolic pathways leading to the production of lactate as a primary end-product of fermentative metabolism.

**Figure 4:**
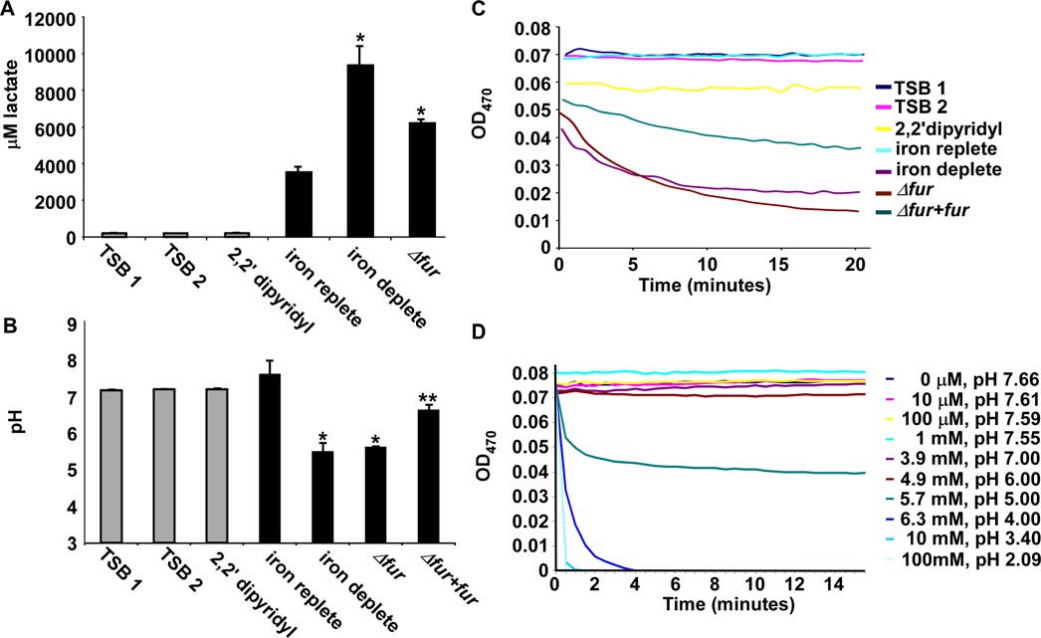
Lactate Produced by Iron-Starved S. aureus Facilitates Iron Release from Transferrin (A and B) pH and lactate concentrations of fresh sterile medium *(TSB 1),* sterile medium after 15 hour incubation at 37 °C *(TSB 2),* fresh sterile medium containing 1mM DIP *(2,2*′ *dipyridyl),* spent medium from wild-type staphylococci grown under iron-replete conditions *(iron replete),* spent medium from wild-type staphylococci grown under iron-starved conditions *(iron deplete),* spent medium from the *Δfur* mutant grown in TSB *(Δfur),* or spent medium from the *Δfur* mutant with full-length *fur* provided in *trans (Δfur+fur)*. Asterisks denote statistical significance as determined by the Student's *t*-test. A single asterisk represents a comparison to iron-replete cultures, while dual asterisks represent a comparison to iron-deplete or *Δfur* cultures. (C) Iron release from transferrin mediated by various media samples. A decrease in optical density signifies a release of iron from transferrin. (D) Iron release from transferrin mediated by various lactate concentrations shown as molar values. The corresponding pH of the media containing the listed lactate concentrations is shown.

To test this hypothesis, we measured the amount of lactate produced by S. aureus after growth in either iron-replete conditions, upon iron starvation elicited by DIP, or upon *fur* inactivation. S. aureus grown under iron-starved conditions produced approximately 3-fold more lactate than S. aureus grown in the presence of iron. Similarly, inactivation of *fur* increased lactate production by approximately 2-fold in iron-replete medium (Figure 4A). To test whether this increase in lactate production contributes to a commensurate decrease in pH, we measured the pH of medium from cultures of iron-replete, iron-deplete, *Δfur,* and *Δfur* containing a full-length copy of *fur* provided in *trans* (*Δfur + fur*). These experiments demonstrated a drop in the pH of the iron-starved culture from 7.2 to 5.2 upon either iron starvation or *fur* inactivation (Figure 4B). Providing *fur* in *trans* complemented the pH decrease of the *Δfur* strain linking the observed decrease in pH to an absence of *fur*. When subjected to identical growth conditions, iron-replete cultures increased pH to close to 8.0 (Figure 4B). From these data, we conclude that S. aureus elaborates a Fur-mediated redirection of central metabolism under iron starvation to increase lactate production and decrease pH.

Transferrin-iron represents a viable iron source to invading bacterial pathogens. In order to utilize transferrin-iron as a nutrient iron source, the iron must be dissociated from transferrin and transported into the bacterium. Free Fe^3+^ is more readily utilized as a nutrient source than transferrin bound iron, and iron is known to dissociate from transferrin upon changes in pH [30]. We hypothesized that the Fur-dependent redirection of central metabolism by iron-starved S. aureus facilitates the release of iron from transferrin through a decrease in local pH. To test this hypothesis, we measured iron release from transferrin mediated by spent medium from either iron-replete, iron-deplete, *Δfur, or Δfur + fur* staphylococcal cultures. We found that medium from iron-starved or *Δfur* staphylococcal cultures significantly increased the rate of iron release from transferrin and this phenotype was partially complemented by providing *fur* in *trans* (Figure 4C). A similar pattern of iron release was observed upon incubation of transferrin in the presence of lactate (Figure 4D). Taken together, these results demonstrate that the Fur-mediated production of lactate by iron-starved S. aureus facilitates the release of iron from host iron-sequestering proteins.

## Discussion

In this study, we have analyzed changes in the cytoplasmic protein profile of *S*. *aureus* upon genetic *(Δfur)* and biochemical (iron chelation, hemin treatment) alterations in iron exposure. Using large-format, high-resolution DIGE with mixed-sample internal standards, we simultaneously surveyed the S. aureus proteome in response to these manipulations *versus* control in quadruplicate to provide for statistical confidence. Overall, 156 protein features of interest, specifying approximately 120 individual proteins (including changes in post-translational modification) were identified by mass spectrometry and placed into functional groups defining Fur-dependent and independent iron regulation as well as hemin-affected proteins.

The hemin-affected proteins were particularly of note because this class of proteins has not previously been characterized in bacterial pathogens, despite the identification of heme-regulated proteins in other bacterial pathogens such as *Corynebacterium diptherhia* [31] and Bordetella sp. [32,33]. The majority of the 21 proteins in this class were decreased upon hemin exposure. However, a few notable exceptions were identified, including a dramatic 45-fold increase of a single protein component (SAV2359) of a putative transporter system which we have named the heme-regulated transporter (HrtAB). This dramatic increase in abundance in the presence of hemin suggests a role for the HrtAB system in heme transport. As a preliminary test of this hypothesis, we individually inactivated *hrtA* and *hrtB* and monitored the ability of these strains to grow in the presence of hemin as a sole iron source. These experiments demonstrated a severe growth restriction on hemin upon inactivation of *hrtAB* and identify the HrtAB system as a critical component of staphylococcal heme metabolism. The Hrt system joins the heme transport system (HtsABC) and iron-regulated surface determinant system, as a third membrane-associated heme transporter [4,8]. The presence of three separate membrane-associated transport systems with roles in heme transit underscores the value of heme metabolism to S. aureus.

The response regulator SaeR was also increased upon exposure to hemin, whereby the modest 1.34-fold increase (*p* = 0.02) may well have profound affects on gene transcription of target proteins. SaeR together with SaeS activates the transcription of several exoproteins including α-hemolysin and β-hemolysin [34], two proteins with potent erythrocyte lysis activity. It is tempting to speculate that the recognition of host heme up-regulates SaeR expression, in turn activating α- and β-hemolysins, which would lead to an increase in local erythrocyte hemolysis and free hemoglobin concentrations. This might represent a positive regulatory circuit used by S. aureus to increase local heme concentrations, and hence nutrient iron availability.

Another noteworthy class of proteins identified in our study was decreased upon inactivation of *fur* in an iron-dependent manner, suggesting a Fur-mediated increase in abundance of these proteins. In gram-negative bacteria, the mechanism for Fur-mediated positive regulation of proteins has been elucidated by elegant studies beginning with the work of Masse et al. [35]. These and other investigations have identified the small regulatory RNA (sRNA) RhyB as being responsible for Fur-dependent protein activation in E. coli [35], Pseuodomonas aueruginosa [36], V. cholera [37], and Shigella flexerni [38]. The targets of RhyB include some of the same genes identified in our study as being positively regulated by Fur, including the TCA cycle enzymes aconitase *(acnA),* fumarase *(fumA),* and succinate dehydrogenase *(sdhCDAB)* [21]. This observation suggests that a similar mechanism of iron-dependent gene regulation is occurring in *S. aureus,* however we were unable to identify any potential homologues to RyhB in any Gram positive bacterial genome using traditional BLAST analyses (unpublished data). S. aureus has been reported to express at least 12 sRNAs with predicted roles in translational regulation through message stability [39]. It is likely that an as-yet-undiscovered sRNA-mediated regulatory system exists in S. aureus responsible for iron homeostasis through targeted mRNA degradation.

Our data indicate that iron starvation leads to the reversible inactivation (or down-regulation) of TCA cycle enzymes including aconitase, the down-regulation of which has been implicated as a survival response to oxidative stress induced during the host-pathogen interaction [40]. In *S. aureus,* downregulation of the TCA cycle through aconitase inactivation prevents maximal expression of the virulence factors lipase, staphylococcal enterotoxin C, and α- and β-hemolytic toxins and therefore alters the interaction between S. aureus and the host [40]. Additionally, inactivation of the TCA cycle or growth in iron-deplete conditions leads to a decrease in production of formylated delta-toxin, a potent neutrophil attractant [41]. Combined, these two factors have led to the suggestion that down-regulation of the TCA cycle may protect against host immune-mediated recognition of infecting S. aureus [41].

We propose a model whereby upon iron starvation, such as would be encountered inside the host, S. aureus up-regulates glycolysis through the release of Fur-mediated repression of glycolytic enzymes. Based on the simultaneous Fur-mediated down-regulation of TCA cycle enzymes, pyruvate does not enter the TCA cycle but instead is acted on by fermentative pathways. We have demonstrated here that four separate branches of fermentative metabolism are down-regulated under iron starvation, which we predict funnels excess pyruvate into acidic fermentative end-products including lactate (Figure 3).

The production of the acidic end-product lactate contributes to a decrease in the local pH of the microenvironment surrounding infecting staphylococci, a hypothesis supported by the observation that the pH of the spent medium from iron-starved or *Δfur* staphylococci is significantly more acidic than that of spent medium from iron-replete cultures (Figure 4B). This overproduction of lactate and subsequent decrease in pH dissociates iron from host iron-sequestering molecules (Figure 4C and 4D). Further, the decrease in the local pH combined with a commensurate decrease in Eh (oxidation reduction potential) would be expected to change the oxidation state of host iron atoms converting the insoluble ferric iron to the more bioavailable ferrous iron. An increase in local ferrous iron concentrations would significantly relieve the iron stress placed on the bacterium and provide a growth advantage to invading staphylococci. The Fur-mediated redirection of central metabolic pathways to increase iron availability is supported by published results showing that the uptake of iron (presented as ^59^FeSO_4_) by S. aureus is twice as great at pH 4.7 as it is at a pH 7.4 [42].

It is possible that additional acidic end-products of fermentative metabolism contribute to the acidification of the culture medium upon growth of iron-starved S. aureus. For instance, the production of formate as a fermentative end-product would contribute to a decrease in the pH of the microenvironment surrounding iron-starved S. aureus. Acetyl-CoA is converted to formate by formate acetyltransferase (PflB), an enzyme that was identified in our proteomic analyses by three separate isoforms exhibiting decreased abundance upon iron starvation or *fur* inactivation (Table 3). These results suggest that formate does not significantly contribute to the acidification of spent medium from iron-starved staphylococcal cultures. However, we did identify a single and separate isoform of PflB that increased expression upon iron starvation (2.03-fold; Table 4). Formate dehydrogenase, which subsequently converts formate to NADH, H^+^, and CO_2_, also exhibited an increase in abundance upon iron starvation (22.32-fold), consistent with the possibility that appreciable amounts of formate are formed by iron-starved staphylococci. We were unable to detect a significant increase in formate accumulation in the medium of iron-starved staphylococcal cultures (unpublished data), suggesting that if formate is being accumulated as a result of iron starvation, it is a transient increase due to catabolism by Fdh.

Although the experiments described here were performed in vitro, the severe iron restriction encountered by S. aureus once inside the host strongly supports an in vivo relevance for these findings. These fundamental changes in metabolic function potentially provide a survival advantage to S. aureus by preventing maximal activation of the immune system while the bacteria struggle to alter its microenvironment to access host iron.

## Materials and Methods

### Bacterial strains and growth conditions.

S. aureus clinical isolate Newman was used in all experiments. Prior to cytoplasmic extraction, bacteria were grown in TSB for 15 h at 37 °C with shaking at 180 rpm. Iron starvation was achieved by addition of 1 mM DIP to the growth cultures prior to inoculation. Hemin treatment was achieved by addition of 10 μM hemin to the growth cultures prior to inoculation. All cultures were incubated in the dark to maintain the integrity of the hemin. To avoid differential gene expression due to growth phase, the cultures were harvested at comparable optical densities during early stationary phase. Newman *Δfur* was created through transduction of the previously created *Δfur* allele from RN4220 [43] to strain Newman with the transducing phage Φ-85 as previously described [10].

S. aureus strain Newman Δ*fur* was complemented by providing a full-length copy of *fur* (SAV1498) under the control of its native promoter in the context of a promoterless pOS1-derived vector. *fur* was PCR amplified from S. aureus Newman genomic DNA using a 5′ primer containing an EcoRI site and a 3′ primer containing a BamHI site. The PCR product was cloned into pCR2.1 (Invitrogen, Carlsbad, California, United States) and excised by digestion with EcoRI and BamHI (New England Biolabs, Beverly, Massachusetts, United States). pOS1 was digested with EcoRI and BamHI and *fur* was inserted, yielding pOS1*fur,* where *fur* is under the control of its native promoter. pOS1*fur* and pOS1 were electroporated into the restriction deficient primary recipient RN4220, after which they were electroporated into appropriate electrocompetent secondary recipient strains (Newman and Newman Δ*fur* for pOS1, Newman Δ*fur* for pOS1*fur*). S. aureus strains harboring plasmids were selected on and grown in either tryptic soy agar or tryptic soy broth containing 10 μg/ml chloramphenicol.

*hrtA* and *hrtB* mutants were obtained from the Phoenix (N) library, clones PhiNE 03177 (SAV2359) and PhiNE 01762 (SAV2360) [44]. The Phoenix mutant isolates are derivatives of the clinical S. aureus isolate Newman that has been mutagenized using the transposon *bursa aurealis* transposon. The exact site of transposon insertions have been determined by DNA sequencing and inactivated genes annotated using the S. aureus Mu50 genome [44]. The *bursa aurealis* insertions in *hrtA* and *hrtB* were transduced into wild-type S. aureus Newman with the transducing phage 85 as previously described [10].

### Preparation of cytoplasmic fractions.

Cytoplasmic extracts were prepared upon completion of 15 h of bacterial growth. Cells were pelleted by centrifugation at 6,000 g for 15 min. Pellets were resuspended in sucrose buffer (100 mM Tris-HCl [pH 7.0], 500 mM sucrose, 100 mM MgCl_2_) and incubated at 37 °C for 45 min in the presence of 1 mg of lysostaphin. Following cell wall digestion, protoplasts were isolated by centrifugation at 13,700 *g* and washed once and resuspended in 20 ml of buffer (50 mM Tris [pH 7.5], 150 mM NaCl, 100 μM phenylmethylsulfonyl fluoride [PMSF]). To lyse the protoplasts, samples were subjected to two rounds of French Press mediated lysis at 20,000 psi. Insoluble material was removed by ultracentrifugation at 100,000 *g* for 45 min. The collected supernatant, representing the cytoplasmic fraction, was used in subsequent analyses. Successful fractionation was confirmed by immunoblot using fraction specific antisera which recognize the cytoplasm (α-IsdG), membrane (α-IsdE), and cell wall (α-IsdB) of S. aureus [10].

### DIGE/MS.

Quadruplicate samples from the four conditions were independently prepared as described above. For each sample, 0.25 mg of protein was separately precipitated with methanol and chloroform [45] and resuspended in 30 μl of lysis buffer (7 M urea, 2 M thiourea, 4% CHAPS, 30 mM Tris, 5 mM magnesium acetate). The NHS-ester dyes Cy2/3/5 were used for the minimal labeling protocol using an internal standard [12,13,46]. Briefly, one-third of each sample (10 μl, 83 μg) was removed and combined into a single tube to comprise the pooled-sample internal standard (1,330 μg total). The remaining two-thirds of each individual sample (20 μl, 167 μg) was individually labeled with 200 pmol of either Cy3 or Cy5, while the pooled-sample was labeled en masse with 1,600 pmol of Cy2. The samples were quenched with 10 mM lysine (2 μl for each 200 pmol) for 10 min on ice, followed by the addition of equal volume 2× rehydration buffer (7 M urea, 2 M thiourea, 4% CHAPS, 4 mg/ml DTT). Pairs of Cy3/Cy5-labeled samples were mixed with an equal aliquot of the Cy2-labeled internal standard according to the schema in Figure 1. Tripartite samples were brought up to 450 μl with 1× rehydration buffer (same as 2× buffer but with 2 mg/ml DTT and 0.5% IPG buffer 4–7) and passively rehydrated into 24-cm 4–7 immobilized pH gradient (IPG) strips for 24 h (total of 500 μg per gel).

All 2D DIGE-associated instrumentation was manufactured by GE Healthcare/Amersham Biosciences (Piscataway, New Jersey). First-dimensional separations were performed on a manifold-equipped IPGphor first-dimension isoelectric focusing unit, and second-dimensional 12% SDS-PAGE was carried out using hand-cast gels that had one plate presilanized (to ensure subsequent accurate robotic protein excision) using an Ettan DALT 12 unit, both according to the manufacturer's protocols. Cy2/3/5-specific 16-bit data files were acquired at 100 μm resolution separately by dye-specific excitation and emission wavelengths using a Typhoon 9400 Variable Mode Imager, and the gels were stained for total protein content with SyproRuby (Molecular Probes/Invitrogen) per the manufacturer's instructions.

The DeCyder v6.5 suite of software tools (Amersham Biosciences/GE Healthcare) was used for DIGE analysis. The normalized volume ratio of each individual protein spot-feature from a Cy3- or Cy5-labeled sample was directly quantified relative to the Cy2-signal from the pooled-sample internal standard corresponding to the same spot-feature. This is performed for all resolved features in a single gel where no gel-to-gel variation exists between the three co-resolved signals. The individual signals from the Cy2-standard were then used to normalize and compare Cy3:Cy2 and Cy5:Cy2 abundance ratios across the eight-gel set, enabling statistical confidence to be associated with each change in abundance or charge-altering post-translational modification using Student's *t*-test and ANOVA analyses (Table S1). Unsupervised PCA and hierarchical clustering was performed using the DeCyder Extended Data Analysis (EDA) module.

Proteins of interest were robotically excised, digested into peptides in-gel with modified porcine trypsin protease (Trypsin Gold; Promega, Madison, Wisconsin, United States) and peptides applied to a stainless steel target using an integrated Spot Handling Workstation per the manufacturer's recommendations. Matrix assisted laser desorption/ionization, time-of-flight mass spectrometry (MALDI-TOF MS) and data-dependant TOF/TOF tandem MS/MS was performed on a Voyager 4700 (Applied Biosystems, Framingham, Massachusetts, United States). The resulting peptide mass maps and the associated fragmentation spectra were collectively used to interrogate S. aureus Mu50 sequences to generate statistically significant candidate identifications using GPS Explorer software (Applied Biosystems, Foster City, California, United States) running the MASCOT search algorithm (http://www.matrixscience.com). Searches were performed allowing for complete carbamidomethylation of cysteine, partial oxidation of methionine residues, and one missed cleavage. Molecular Weight Search (MOWSE) scores, number of matched ions, number of matching ions with independent MS/MS matches, percent protein sequence coverage, and correlation of gel region with predicted MW and pI were collectively considered for each protein identification (all data are presented in Table S1).

### Growth curve assays.

S. aureus cultures were grown overnight under low-iron conditions by inoculating strains in RPMI supplemented with 1% casamino acids plus 200 μM DIP. Overnight cultures were then subcultured in NRPMI (Chelex- treated RPMI) containing 500 μM DIP, and inoculated into NRPMI+ (NRPMI containing 25 μM ZnCl_2_, 25 μM MnCl_2_, 1 mM MgCl_2_, 100 μM CaCl_2_) supplemented with 500 μM DIP, and 20 μM iron sulfate or 10 μM hemin as indicated. Cultures were grown at 37 °C with aeration in a round-bottom 96-well plate and bacterial growth was monitored by increase of absorbance (O.D._600_) over time.

### Measuring pH, transferrin-Fe release, and lactate.

Bacteria were grown in 10 ml of tryptic soy broth (TSB) in a 50-ml flask for 15 h at 37 °C with 180 rpm shaking. All strains containing derivatives of pOS1 were grown in the presence of 10 μg/ml chloramphenicol to ensure successful maintenance of the plasmid and to normalize growth conditions. Iron was chelated from the media by adding DIP to a final concentration of 1 mM. After 15 h, the cultures were centrifuged and the supernatants were collected. All pH values were measured using a S20 SevenEasy pH meter (Mettler Toledo).

Measuring the release of iron from transferrin was performed as previously described [47]. Iron-bound transferrin exhibits an absorption peak at 470 nm. As iron dissociates from transferrin the intensity of the peak at 470 nm absorption decreases. Absorption at 470 nm was measured every 30 s for 15 min upon introduction of the samples. Transferrin stock solutions of 400 μM were prepared by suspending human transferrin (Sigma, St. Louis, Missouri, United States) in distilled water. Transferrin stock solutions were added at a final concentration of 40 μM to all samples. All absorption readings were measured using a Cary 100 UV-Vis spectrophotometer (Varian).

To measure lactate concentrations, bacteria were grown in 5 ml (TSB) for 15 h at 37 °C with 180 rpm shaking. Lactate levels were measured using a Lactate Assay Kit according to manufacturer's recommendations (Department of Biochemistry at The State University of New York at Buffalo). This kit measures the lactic acid–dependent production of formazan which exhibits an absorption maximum of 492 nm and is directly proportional to the concentration of lactate.

## Supporting Information

Table S1 Master Table of All DIGE Profiling and MS Database Search Results (671 KB XLS)

## Abbreviations

2D: two-dimensional
DIGE: difference gel electrophoresis
DIP: 2,2′-dipyridyl
Fur: ferric uptake regulator
MS: mass spectrometry
PCA: principal component analysis
sRNA: small regulatory RNA
TCA: tricarboxylic acid
TSB: tryptic soy broth
